# The effect of *Chlorella pyrenoidosa* supplementation on immune responses to 2 days of intensified training

**DOI:** 10.1007/s00394-017-1525-9

**Published:** 2017-08-19

**Authors:** Corinna Chidley, Glen Davison

**Affiliations:** Endurance Research Group, School of Sport and Exercise Sciences, The Medway Campus, University of Kent, Chatham Maritime, ME4 4AG UK

**Keywords:** Cycling, Single-celled microalgae, Algae, IgA, Exercise, Nutrition

## Abstract

**Purpose:**

Periods of intensified training are associated with immune disturbances, The aim was to investigate the effects of supplementation with* Chlorella pyrenoidosa* (*Chlorella*) on secretory IgA (sIgA) responses to 2 days intensified training.

**Methods:**

Twenty-six subjects (age 29.1 ± 8.7 years; VO_2max_ 53.7 ± 11.7 ml kg min^−1^) provided resting saliva samples for determination of sIgA, at baseline (week-0) and following 4, 5, and 6 weeks (weeks-4, -5, -6) of daily supplementation with 6 g/day *Chlorella* (*n* = 13) or placebo (PLA, *n* = 13). During week-4 a 2-day intensified training period was undertaken [morning and afternoon sessions each day, respectively: VO_2max_ test; high-intensity interval training (HIIT, 3 × 30 s Wingate sprints); 90 min at ~60% VO_2max_; 3 × 30 s HIIT].

**Results:**

*Chlorella* increased resting sIgA secretion rate (trial × time, *P* = 0.016: no change with PLA but increases with *Chlorella* at week-4, week-5 and week-6, *P* = 0.020, <0.001, and 0.016). PLA vs *Chlorella*: week-0 = 54 ± 33 vs 57 ± 37 µg/min; week-4 = 54 ± 35 vs 83 ± 57 µg/min; week-5 = 63 ± 46 vs 98 ± 47 µg/min; week-6 = 58 ± 35 vs 85 ± 59 µg/min. Minimal acute changes in sIgA were seen in response to individual exercise bouts, but it was higher at some times in the *Chlorella* group (for bouts 2 and 3).

**Conclusion:**

Supplementation with *Chlorella* has beneficial effects on resting sIgA, which might be beneficial during periods of intensified training.

**Electronic supplementary material:**

The online version of this article (doi:10.1007/s00394-017-1525-9) contains supplementary material, which is available to authorized users.

## Introduction

It is widely reported that athletes engaged in regular prolonged and/or strenuous exercise (e.g. endurance athletes) have a higher than normal incidence of upper respiratory tract infection (URTI) symptoms (URS), which may be related to an exercise-induced impairment of immune function (immunodepression) [1, 2]. This is thought to be related to a high total training load and also the way training is distributed or periodised. In support of this, Svendsen et al. [3] have recently shown that rapid changes in training load (i.e. increasing too quickly) are better predictors of URS risk than total training load alone, which agrees with previous research in this area [4, 5]. However, many athletes must train intensively to be successful. It is also common for athletes to undergo short periods of intensified training (e.g. when training load is rapidly increased intentionally) with the aim of enhancing physiological adaptation and performance. For this reason, it is inevitable that they will have an increased risk of immunodepression and URTI susceptibility during and after these times. Hence, any nutritional intervention that can minimise these negative effects will be of benefit to such athletes.

Many nutritional interventions have been investigated as potential countermeasures to exercise-induced immunodepression, but the evidence for many is limited [6–8]. One food supplement commonly used in Japan, which has been suggested to have beneficial effects on immune function, is the freshwater single-celled microalgae *Chlorella pyrenoidosa*. The effects of *Chlorella pyrenoidosa*, as a nutritional supplement, on immune function have been widely investigated. This includes findings of improved aspects of mucosal immunity [9–11], enhanced Th1 immune responses [12–15], and improved natural killer (NK) cytotoxic activity (NKCA, [13]). However, there are relatively few studies of humans in vivo, and fewer still on athletes and those with high physical activity levels.

*Chlorella pyrenoidosa* contain a number of vitamins, minerals, and amino acids (see Table S1). However, the doses consumed with typical daily intakes (e.g. 6 g/day in total) are unlikely to be of benefit to individuals who are not nutritionally deficient (so many athletes will already be abundant in these nutrients from their normal diet). The benefits seen with *Chlorella pyrenoidosa* supplementation, however, are more likely caused by other substances which have bioactive properties and are bioavailable after consumption. It has been shown that many of the polysaccharides and glycoproteins (or protein/polysaccharide complexes) found in chlorella are responsible for the immunostimulating properties [16–18]. Indeed, *Chlorella pyrenoidosa* supplementation has been shown to increase both sIgA concentration and secretion rate [9, 11] when consumed daily (5–6 g/day) for 4–8 weeks in healthy humans. One previous study of athletes observed protection against the detrimental effects of an intensified training period on salivary sIgA levels [10]. In that study 10 female kendo athletes were supplemented with either 6 g/day *Chlorella* or placebo for 4 weeks ahead of a 5-day training camp. Salivary sIgA concentration and secretion rates were maintained near baseline levels throughout the duration of the training camp in the *Chlorella* group compared to the significant reductions observed in the control group. The findings of this study, although in only a limited cohort of athletes, demonstrates that chlorella may help attenuate immunodepression observed following intensified periods of training. Furthermore, none of the previous studies have assessed the response to individual sessions in the training period. Hence, the purpose of this study was to investigate the effects of *Chlorella pyrenoidosa* supplementation on immune responses to a 2-day intensified training period.

## Methods

### Ethical approval

The study was conducted in accordance with the Declaration of Helsinki and approved by the University of Kent’s Research Ethics Advisory Group. All subjects were informed both verbally and in writing of the nature and risks of the experimental procedures after which written consent was obtained.

### Study design and subjects

Thirty-four healthy and physically active subjects with experience of exercise training (i.e. at least three times per week of planned physical exercise) volunteered for the study (27 males and 7 females). Subjects were excluded from participation if they were using nutritional supplements or medication, or if they had given blood, received vaccinations, or suffered an infection within 1 month of the study commencing. All subjects provided written informed consent before participation.

Subjects were randomised to receive 6 g/day chlorella (SC, *n* = 17, 3 female) or placebo (PLA, *n* = 17, 4 female) daily. Treatments were administered in a double-blind manner. A total of 8 subjects were lost to follow-up/withdrew before completion of the study due to issues unrelated to the study (lack of time or sporting injury) leaving *n* = 13 (4 female) in the placebo group and *n* = 13 (1 female) in the chlorella group (however, covariate analysis found no influence of sex on any of the measures so all analyses were completed with combined male and female results). Furthermore, physical characteristic profiles and indicators of physiological fitness were similar between groups (PLA vs SC, respectively: age 31 ± 9 vs 27 ± 9 years, *P* = 0.204; height 171 ± 7 vs 175 ± 7 cm, *P* = 0.170; weight 70.8 ± 12.5 vs 71.8 ± 9.4 kg, *P* = 0.809; VO_2max_ 50.9 ± 9.9 vs 57.7 ± 13.2 ml kg^−1^ min^−1^, *P* = 0.153; gas exchange threshold as % of VO_2max_ 46.3 ± 5.5 vs 44.0 ± 8.0%, *P* = 0.410).

### Supplements

In line with previous research, the final dose was 6 g per day (30 tablets) of *Chlorella pyrenoidosa* tablets (Sun Chlorella ‘A’ tablets, Sun Chlorella Corporation, Kyoto, Japan) or placebo [9–11] (see Table 1 for nutritional composition). The placebo tablets were also provided by Sun Chlorella Corporation and were indistinguishable from the Chlorella tablets. Supplements were provided in sealed foil pouches with a blinding code that was held by the manufacturer and not revealed until after all analyses had been completed. The dose was gradually increased over the first 3 days starting with a total of 10 tablets per day on day 1, 20 tablets per day on day 2 and 30 tablets per day from day 3 and all remaining days during the supplementation period. Subjects were advised to swallow their tablets with water, consuming half alongside their breakfast and half alongside their evening meal for the duration of the study.

**Table 1:** Nutritional values of placebo and SunChlorella A tablets

	Placebo	*Chlorella*
Energy, kcal/6 g	24.36	23.22
Moisture, g/6 g	0.19	0.29
Protein, g/6 g	0.12	3.28
Fat, g/6 g	0.35	0.71
Total carbohydrate, g/6 g	5.21	1.28
Sugar, g/6 g	5.14	0.53
Dietary fibre, g/6 g	0.07	0.75
Ash, g/6 g	0.13	0.43

### URTI questionnaires

Subjects completed the Jackson common cold questionnaire [19] and episodes of illness were identified and scored as previously described [20].

### Trials

Subjects reported to the laboratory between 06:30 and 08:30 h following an overnight fast. Baseline saliva samples were collected after 10 min of seated rest, and at least half an hour after waking [21]. Subjects were provided with the first 4 weeks of supplementation, along with illness questionnaires to be completed daily. After 4 weeks of supplementation they returned for the 2-day intensified training period. Subjects continued to take their supplement and record daily illness diaries, for a further 2 weeks after the week in which the intensified training took place. Resting saliva samples were collected pre-supplementation (Baseline), after 4 weeks of supplementation, before the intensified training (week-4-day 1), during the intensified training (resting, pre-exercise on day 2: week-4-day 2) and 1, and 2 weeks after the intensified training (week-5 and week-6, respectively). Additional samples were also obtained immediately before (Pre-Ex), after (Post-Ex) and 1 h after (1 h Post-Ex) each training session of the intensified period.

### Exercise trials

#### 2-day intensified training period

After 4 weeks of daily supplementation subjects undertook 4 exercise bouts in 2 days, one in the morning and one in the afternoon of each day (sessions 1–4). Session 1 was an incremental maximal oxygen uptake test; session 2 a high-intensity interval training (HIIT) session; session 3 a prolonged endurance ride; and session 4 a second HIIT session.

*Day 1* On the first day subjects reported to the laboratory between 06:30 and 08:30 h following an overnight fast. Height and weight were recorded and subjects were fitted with a heart rate monitor (Polar Electro, Kempele, Finland), and facemask (Cortex Biophysik GmbH, Leipzig, Germany) connected to a breath-by-breath gas analyser (MetaLyzer 3BR2, Cortex Biophysik GmbH, Leipzig, Germany). Subjects completed a ramped incremental $\dot{V}{\text{O}}_{{2\max }}$ test until volitional exhaustion on an electronically braked cycle ergometer (Excalibur Sport, Lode, Groningen, the Netherlands), beginning with 3 min of unloaded cycling before initiation of a continuous increment in work load of 30 W/min until volitional exhaustion. Subjects’ $\dot{V}{\text{O}}_{{2\max }}$, and first gas exchange threshold were identified from the test data collected so that 25% of the difference between these values (Δ) could be calculated (the power output associated with 25% Δ was used for the prolonged endurance bout the next day). Subjects were allowed to return home following post-exercise sample collections, and then reported to the laboratory again between 16:00 and 18:00 h having refrained from eating within the hour preceding the test. A friction-braked cycle ergometer (Monark Ergomedic 874e; Monark Exercise AB, Vansbro, Sweden) was prepared and the basket loaded with 7.5% of the subject’s body mass. Subjects then performed a 5-min warm-up at 70 rpm with the basket suspended (with a 5-s sprint against 7.5% body weight resistance at 3 min). After 2 min of passive recovery seated on the ergometer, subjects performed three 30-s maximal (‘all-out’) sprints with 90-s recovery between each. Subjects were allowed to bring their cadence up to 70 rpm before the basket was released, at which point they were required to sprint maximally (all-out effort) for 30 s. Following each sprint, subjects were asked for their rating of perceived exertion (RPE) for the sprint just completed [22].

*Day 2* Subjects once again reported to the laboratory between 06:30 and 08:30 h following an overnight fast. Subjects were fitted with a heart rate monitor (Polar Electro, Kempele, Finland). Subjects cycled for 90 min at 25% Δ, the intensity of which was validated within the first 10 min by monitoring V̇O_2_ responses, and the intensity adjusted accordingly (subjects were monitored continually during the first 10 min using the online system; MetaLyzer 3BR2, Cortex Biophysik GmbH, Leipzig, Germany); expired gas was collected into Douglas bags (Plysu Industrial, Ltd., Milton Keynes, UK) every 20 min thereafter for analysis of F_E_O_2_ and F_E_CO_2_ using a gas analyzer (Servomex, West Sussex, UK) and volume measured using a dry gas meter (Harvard Apparatus, Kent, UK) to determine gas exchange variables as previously described [23], RPE and heart rate (HR) were recorded every 10 min. A water bolus of 2 mL kg^−1^ body mass was provided every 15 min. Following post-exercise samples subjects were once again allowed to leave the laboratory and return later the same day between 16:00 and 18:00 hours having refrained from eating in the hour preceding exercise. Subjects then completed an identical HIIT protocol to the one undertaken the previous day.

### Sample collection and analytical methods

Saliva samples were collected as previously described [24]. Briefly, timed, unstimulated whole saliva was obtained whilst subjects were seated, with the head tilted slightly forward and making minimal orofacial movement. They were asked to swallow to empty the mouth, before whole saliva was collected by passive dribble into a pre-weighed sterile tube for an initial period of 2 min. If insufficient saliva was obtained after the initial 2-min period the process was repeated. Prior to samples being collected, subjects rinsed their mouth with fresh tap water and remained seated for 10 min. The tube was re-weighed after collection of the sample so that saliva volume (and hence flow rate) could be estimated. Tubes were weighed to the nearest 0.1 mg (saliva density was assumed to be 1.00 g ml^−1^). Saliva samples were centrifuged in a micro-centrifuge at 17,000×*g* for 5 min, and the supernatant stored in aliquots at −80 °C until analysis of sIgA by sandwich ELISA (modified from Leicht et al. [25], but using a capture antibody specific to secretory IgA, I-6635, Sigma-Aldrich, St. Louis, MO, USA).

### Statistics

All results are presented as mean (SD). An alpha-priori level of 0.05 was set for determination of statistical significance. Normality of the distribution of data was assessed using the Shapiro–Wilk test. Normally distributed variables (subject characteristics, exercise trial physiological responses, illness duration, and symptom score) were compared using independent samples t-tests. Number of illness episodes were compared using the Chi-squared test with the Fisher’s exact test. Variables that were not normally distributed (and could not be normalised via transformation) were assessed with appropriate non-parametric tests. Mann–Whitney *U* tests were undertaken for age; RPE of the 90-min trial; and severity of illness. All other variables were compared (across time, between groups) using two-way mixed ANOVA. Where necessary, post hoc follow-up was conducted with Holm–Bonferonni corrected t-tests. All tests were carried out using SPSS Version 22.0 (IBM Corp, Armonk, NY, USA). As previously mentioned, due to different numbers of females in each group (after drop-out from the original *n* = 34) covariate analysis was performed, revealing no influence of sex on the between-group comparisons and so all analyses are reported for males and females combined (comparing only supplementation group, SC vs PLA).

## Results

### Physiological and perceptual responses to exercise bouts

The results from the incremental VO_2max_ test are reported above in the “Study design and subjects” section of the “Methods”. In addition, the mean duration of each test (total exercise time to the point of volitional exhaustion) was similar between groups (PLA 13.1 ± 2.0 min vs SC 14.4 ± 1.8 min, *P* = 0.109).

Physiological and perceptual responses related to the demands of the 90-min continuous exercise trial were similar between groups (mean values for the bout in PLA vs SC, respectively: heart rate 133 ± 12 vs 138 ± 15 beats/min, *P* = 0.378; RPE 13 ± 1 vs 13 ± 1, *P* = 0.762; trial VO_2_ 2.2 ± 0.4 vs 2.3 ± 0.5 L min^−1^, *P* = 0.190; respiratory exchange ratio (RER) 0.95 ± 0.07 vs 0.92 ± 0.04, *P* = 0.344; exercise intensity as % of VO_2max_ 60.5 ± 5.9 vs 60.1 ± 10.4%, *P* = 0.979).

For the HIIT sessions no physiological measures were recorded, but subjects were instructed to give maximal and ‘all-out’ effort (without any pacing and not holding back anything for later in the test) for each sprint repetition. Subjects reported a maximal RPE of 20 following each sprint.

### Salivary immune markers

#### Resting sIgA

**Fig. 1:**
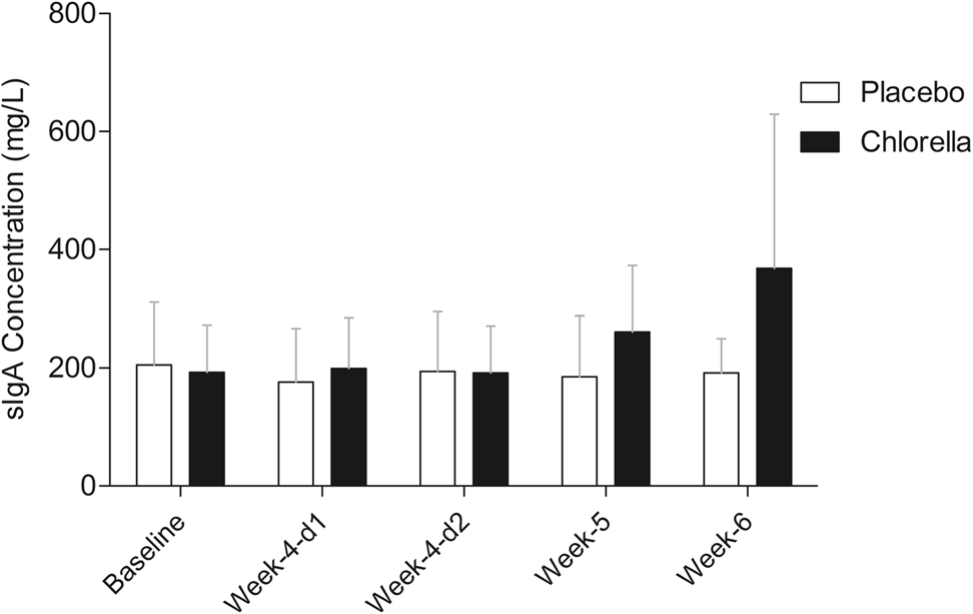
Resting saliva sIgA concentration over study period

**Fig. 2:**
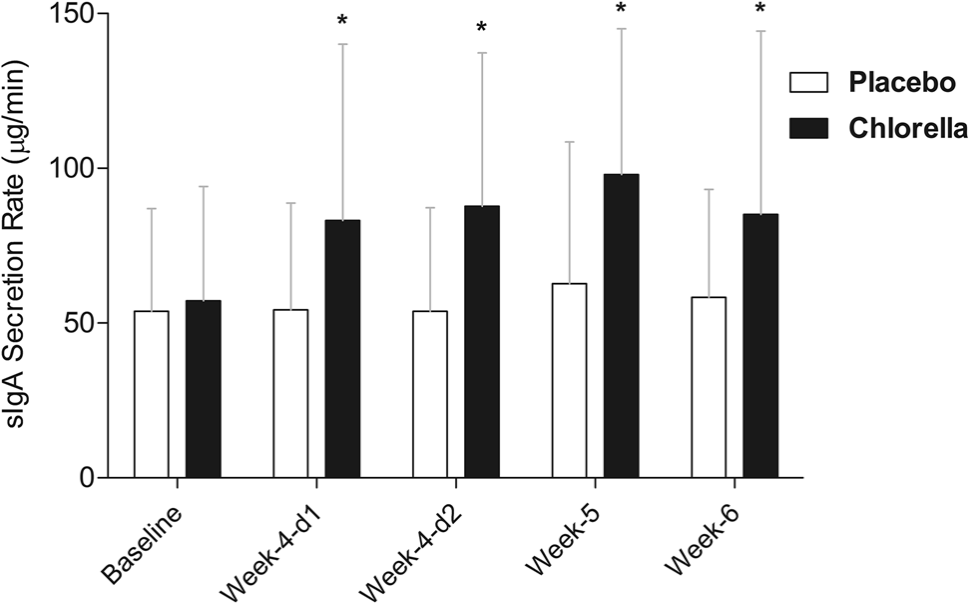
Resting saliva sIgA secretion rate over study period. *Asterisk* (*P* < 0.05) indicates significantly higher than baseline (post hoc follow-up for time: each group analysed separately due to significant group × time-point interaction)

Resting salivary IgA concentration (Fig. 1) showed a significantly different pattern of change over the course of the study (2-way mixed ANOVA trial × time interaction *P* = 0.022). Post hoc analysis revealed no changes in the PLA group and a significant time effect (*P* < 0.001) in the SC group (but when followed with further post hoc tests this only revealed a trend for an increase in the weeks after intensified training (week-5 *P* = 0.078, and week-6 *P* = 0.056). Resting sIgA secretion rate (Fig. 2) showed a significantly different pattern of change over the course of the study (trial × time interaction *P* = 0.016). Post hoc analysis revealed no changes in the PLA group and a significant time effect (*P* = 0.002) in the SC group, which was due to an increase over baseline values at the week-4-day 1 (*P* = 0.020), week-4-day 2 (*P* = 0.006), week-5 (*P* < 0.001), and week-6 (*P* = 0.016) time points.

#### Responses to exercise

During the intensified-training period, there were significant decreases in salivary sIgA concentration in response to some of the exercise bouts (time effects for bouts 1–4: *P* < 0.001, *P* < 0.001, *P* = 0.007, and *P* < 0.001, respectively), but SC did not influence this (trial × time interaction, all *P* > 0.05) (Table 1). When normalised against flow rate (i.e. secretion rate) there was no trial × time interaction for exercise bouts 1 or 4 (*P* = 0.066, *P* = 0.181, respectively). However, there was a significant trial × time interaction for exercise bouts 2 (*P* = 0.016) and 3 (*P* = 0.017). For bout 2 (1st high-intensity interval training session) post hoc analysis revealed no change in the PLA group (*P* = 0.075) but a significant time effect in the SC group (*P* = 0.003). Further post hoc analysis showed an increase above baseline at the pre-exercise (*P* = 0.009) and post-exercise (*P* = 0.010) times. For exercise bout 3 (90 min ride) post hoc analysis revealed no change in the PLA group (*P* = 0.996) but a significant time effect in the SC group (*P* < 0.001). Further post hoc analysis showed an increase above baseline at the pre-exercise (*P* = 0.023), post-exercise (*P* = 0.005), and 1 h post-exercise (*P* = 0.004) times (see Table 2).

**Table 2:** Acute responses to each exercise bout during the 2-day intensified training period

	Baseline	Bout 1 (Day 1 morning): VO_2max_ test			Bout 2 (Day 1 afternoon): HIIT			Bout 3 (Day 2 morning): 90 min SS			Bout 4 (Day 2 afternoon): HIIT		
		Pre-Ex	Post-Ex	1 h Post-Ex	Pre-Ex	Post-Ex	1 h Post-Ex	Pre-Ex	Post-Ex	1 h Post-Ex	Pre-Ex	Post-Ex	1 h Post-Ex
sIgA (mg/L)				**	**		**			*	*		*
PLA	205 (106)	176 (90)	197 (85)	148 (62)	131 (49)	201 (109)	141 (124)	194 (101)	168 (57)	126 (60)	135 (73)	197 (100)	147 (81)
SC	193 (79)	199 (86)	266 (128)	123 (65)	151 (53)	215 (194)	137 (79)	192 (79)	179 (69)	157 (60)	148 (66)	248 (104)	169 (114)
sIgA secretion rate (µg/min)													
PLA	53.8 (33.2)	54.3 (34.5)	66.6 (80.6)	73.3 (73.1)	58.6 (41.1)	45.5 (36.9)	66.6 (73.3)	53.8 (33.5)	52.2 (27.6)	53.7 (39.2)	53.1 (32.8)	46.3 (21.7)	75.6 (41.5)
SC	57.2 (36.9)	83.1 (57.0)	88.7 (67.1)	82.2 (59.1)	**99.9 (82.1)** ^**b**^	**84.3 (66.4)** ^**a**^	92.6 (97.2)	**87.7 (49.6)** ^**a**^	**77.3 (41.0)** ^**b**^	**87.2 (43.3)** ^**b**^	80.3 (37.3)	72.6 (62.9)	124.6 (128.9)

Values are mean (SD)

The* P* values for the items in bold are stated in the text above

*HIIT* high-intensity interval training session; 90 min, *SS* steady-state endurance ride

* (*P* < 0.05) and ** (*P* < 0.01) indicate significantly lower than baseline (post-hoc follow-up for time: both groups pooled due to no group × time-point interaction)

^a^ (*P* < 0.05) and ^b^ (*P* < 0.01) indicate significantly higher than baseline (post-hoc follow-up for time: each group analysed separately due to significant group × time-point interaction)

### URS reports

There was no difference between groups (*P* = 1.000) in the number of URS episodes suffered in the 2 weeks following the training, with 31% of subjects in each group reporting an episode. The duration of URS episodes was also not different between groups (5 ± 2 days PLA vs 8 ± 5 days SC, *P* = 0.333). There was also no difference between groups for mean severity ratings (1.8 ± 0.5 PLA vs 2.0 ± 1.2 SC, *P* = 0.711) or total symptom scores (10.5 ± 7.0 PLA vs 21.5 ± 13.0 SC, *P* = 0.187) of URS episodes.

## Discussion

The primary aim of the current study was to assess the effects of chlorella supplementation on salivary sIgA in response to a 2-day intensified training period. The main findings were that daily supplementation with SC for 4 weeks before (and 2 weeks after) the 2-day intensified training period increased resting sIgA concentration (by week-5) and secretion rate (by week-4). There were no effects on acute responses of sIgA concentration to each of the exercise bouts during the intensified training period, but sIgA secretion rate was increased during some components of the training period in the SC compared to PLA group. There were no differences, however, between PLA and SC groups in self-reported URS incidence or symptom duration.

The acute decreases in salivary sIgA concentration seen after some of the exercise training sessions are in line with some previous studies on intensive interval training protocols [26] but not others [24] in which no acute decreases were seen for sIgA. On the other hand, the lack of an acute effect of each bout on salivary sIgA secretion rate differs from Hall et al. [26] but agrees with Davison et al. [24]. Few studies, however, have assessed the acute responses to multiple exercise sessions over a short time period (e.g. intensified training or training camps). Papacosta et al. [27] and Otsuki et al. [10] have shown decreases in resting sIgA levels over longer periods of intensified training/training camps (i.e. 1–2 weeks in duration), which differs to the present study in which no decreases in resting sIgA concentration or secretion rate were seen during (week-4-day 2) or shortly after (week 5), but this is possibly due to the shorter intensified training period in the present study. The previous studies did not assess the response to individual sessions in the training period, however, which represents a novel aspect of the current study. sIgA is one of the body’s first lines of defence against pathogens entering through the oral cavity. A decrease in sIgA has been shown to be related to an increased risk of developing URTI and the associated symptoms (URS). For this reason, it was hypothesised that any increase in sIgA would translate to a reduced risk of URS (lower incidence of self-reported episodes), in line with previous studies on intensified periods of training/training camps or long-term monitoring of athletes (e.g. [28–31]). However, a limitation of the present study is that the relatively modest sample size (*n* = 26) combined with the relatively low number of total URS episodes experienced during the study period (31% incidence rate) may limit our ability to comprehensively determine the effects on URTI/URS. Furthermore, resting sIgA levels remained relatively stable in the PLA group and decreases were only evident acutely after some, but not all, of the exercise training sessions. This maintenance of sIgA defences, close to baseline levels, in the subjects of this study may mean that URTI/URS risk was not adversely affected to a great extent even with the intensified training period. Despite this, our findings (in combination with those from previous research) do show some benefits from SC in terms of increases, above baseline levels, in salivary sIgA. Importantly, resting sIgA concentration was not significantly increased before or during the intensified training period, but appeared to be increasing in the weeks after. It is possible therefore that more benefit would be evident with a longer supplementation period (e.g. if the intensified training was commenced after 5 or 6, rather than 4, weeks of supplementation) and it is feasible that this would also translate to a greater effect on URS reports. However, this will require further study.

The most likely mechanisms for the increase in salivary sIgA observed after 4–5 weeks of supplementation with *Chlorella* are via the immunostimulating properties of compounds found in chlorella such as specific polysaccharides and glycoproteins or protein/polysaccharides complexes [16–18]. In particular, Kralovec et al. [18] identified relatively high molecular weight (>100 kDa) protein/polysaccharide complexes and polysaccharides as being responsible for the immunostimulatory effects of *Chlorella pyrenoidosa* in vitro. They identified glutamic acid and aspartic acid as the major amino acid constituents of the protein/polysaccharide complexes (~22–26%) with galactose (~22–50%), rhamnose (~18–40%) and arabinose (~14–26%) as the main constituent monosaccharides found in the polysaccharides and protein/polysaccharide complexes with the greatest immune stimulating activity. This included effects on B cell stimulation and proliferation, which could explain the beneficial effects seen in humans in vivo, including antibody response to influenza vaccination (e.g. [32]) and aspects of mucosal immunity as observed in the present study and previous research (e.g. [9–11]). However, it is not possible to determine the precise mechanisms and we can only speculate at this stage. Indeed, *Chlorella* does contain an abundance of nutrients, many of which could influence immunity in athletes. However, in line with previous suggestions [9, 10] the specific doses of each of these nutrients are unlikely to be responsible for the effects we have observed on sIgA in a healthy young adult population with no known dietary deficiencies. As such, we suggest that it is the compounds such as polysaccharides and protein/polysaccharide complexes (as identified by Kralovec et al. [18]) that are responsible for the effects observed in the present study. This will require further study (including the determination of the bioavailability from orally consumed *Chlorella* and monitoring levels of these substances in plasma and/or immune cells after ingestion, but this was beyond the scope of the current investigation).

### Limitations

One limitation of this study is that we did not measure VO_2max_ pre-supplementation, so it is not possible to see the effect of SC supplementation on these parameters. This study was not designed to measure cardiovascular responses to supplementation however, but rather the immunological responses to a controlled period of intensified training (i.e. with all participants exposed to the same relative training demand). We also did not record physiological responses during HIIT sessions to ensure that the exercise intensity was comparable between PLA and SC groups. However, we are confident that subjects produced a maximal effort (and hence the same relative demand) for these sessions based on the maximal RPE values expressed following all HIIT sprints.

In summary, daily supplementation with SC was able to increase salivary sIgA concentration and secretion rate at rest. Together with previous research there is now substantial evidence to show that SC can enhance salivary sIgA; however, in the present study it appears that a longer supplementation period may be required in order to translate to protection against URTI and reduced URS reports.

## Electronic supplementary material

Below is the link to the electronic supplementary material.

Supplementary material 1 (XLSX 12 kb)
